# Biodiversity loss in a Mediterranean ecosystem due to an extreme warming event unveils the role of an engineering gorgonian species

**DOI:** 10.1038/s41598-019-41929-0

**Published:** 2019-04-11

**Authors:** Jana Verdura, Cristina Linares, Enric Ballesteros, Rafel Coma, María J. Uriz, Nathaniel Bensoussan, Emma Cebrian

**Affiliations:** 1 Institut d’Ecologia Aquàtica, Facultat de Ciències, Universitat de Girona, Campus Montilivi, 17071 Girona, Spain; 2 Centre d’Estudis Avançats de Blanes-CSIC, Accés Cala Sant Francesc 14, 17300 Blanes, Girona Spain; 3 Department of Evolutionary Biology, Ecology and Environmental Sciences, Institut de Recerca de la Biodiversitat (IRBIO), University of Barcelona, Av. Diagonal 643, 08028 Barcelona, Spain; 4 Institut Ciències del Mar, CSIC, Barcelona, Spain; 5 Aix Marseille University, Université de Toulon, CNRS, IRD, MIO, Marseille, France

## Abstract

Stochastic perturbations can trigger major ecosystem shifts. Marine systems have been severely affected in recent years by mass mortality events related to positive thermal anomalies. Although the immediate effects in the species demography affected by mortality events are well known, information on the mid- to long-term effects at the community level is much less documented. Here, we show how an extreme warming event replaces a structurally complex habitat, dominated by long-lived species, by a simplified habitat (lower species diversity and richness) dominated by turf-forming species. On the basis of a study involving the experimental manipulation of the presence of the gorgonian *Paramuricea clavata*, we observed that its presence mitigated the effects of warming by maintaining the original assemblage dominated by macroinvertebrates and delaying the proliferation and spread of the invasive alga *Caulerpa cylindracea*. However, due to the increase of sediment and turf-forming species after the mortality event we hypothesize a further degradation of the whole assemblage as both factors decrease the recruitment of *P.clavata*, decrease the survival of encrusting coralligenous-dwelling macroinvertebrates and facilitate the spreading of *C. cylindracea*.

## Introduction

Climate change is impairing ecosystems around the world by affecting the phenology, physiology and ecological interactions of key species, triggering shifts in their distributions, and modifying community composition, structure and dynamics^1–3^. In addition to warming, another consequence of climate change is an increase in the frequency and intensity of extreme climatic events, such as stochastic infrequent perturbations^4–6^ that can drive ecosystem shifts^7,8^. In fact, extreme climatic events are reported to represent greater impacts on natural ecosystems than the progressive temperature increase derived from global warming^9,10^. In comparison to terrestrial ecosystems, where the negative effects of extreme events have been widely documented^11,12^, the responses of marine ecosystems to these events are far less reported and are more poorly understood (but see^7,8,13,14^).

Positive thermal anomalies are likely the major extreme climatic events in marine and oceanic ecosystems^15^. Mass mortality episodes and diseases linked to thermal anomalies have increased during the last few decades^16,17^. However, to date, mass mortality events have only been documented at the species and population levels, especially in engineering species, such as hard corals, gorgonians and sponges^18–21^. The delayed direct and indirect (e.g. through engineering species loss) effects of thermal anomalies at the community level are much less studied^7,8,14^. Moreover, engineering species loss is blamed to facilitate invasions^15,22,23^, which can be fostered if the whole assemblage is affected by thermal stress.

The unpredictability of the occurrence of thermal anomalies and the inherent complexity of *in situ* manipulative experiments have hampered studies on the role of engineering species in protecting entire assemblages from warming events as well as on the evaluation of their contribution (direct and indirect) to reduce the effects (if any) of thermal anomalies at the community level.

The northwestern Mediterranean Sea has been severely affected by mass mortality events of several benthic invertebrates coupled with high-temperature conditions in recent decades^21,24–29^. Additionally, the Mediterranean Sea is especially prone to marine invasions^30^, being one of the most affected areas by the spread of invasive species worldwide^31,32^. One of the Mediterranean habitats most disturbed by both thermal anomalies and invasive species are coralligenous outcrops^22,26,33,34^. Coralligenous assemblages represent highly diverse and structurally complex habitats unique to the Mediterranean Sea^33^. The engineering species that compose this habitat show slow growth and low recruitment rates, which results in a high vulnerability to strong disturbances^35–38^. The red gorgonian *Paramuricea clavata* (Riso, 1826), is one of the most paradigmatic engineering species thriving in coralligenous outcrops^33^. This species can be severely affected by thermal anomalies^10^, with mortalities that can reach up to 80% of the colonies^21^, and by invasive algae that strongly limit their recovery^22^.

**Figure 1:**
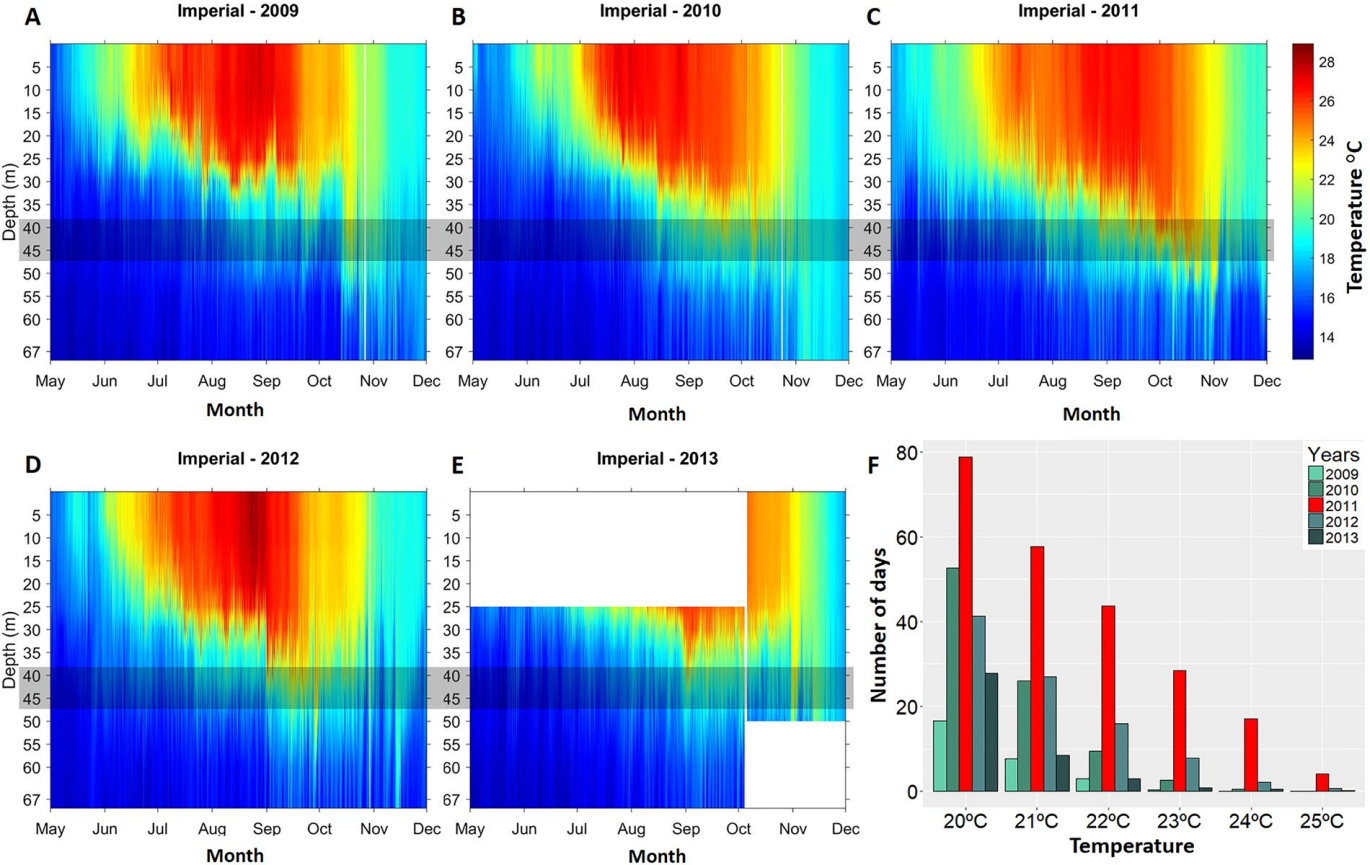
(**A**–**E**) Stratification temperature maps showing the inter-annual variability of the temperature at the study site for the years 2009 to 2013. A grey band is overlaid on the target coralligenous assemblage depth. (**F**) Total time (represented in days) per year that temperature at 40 m depth exceeds the different temperature thresholds.

**Figure 2:**
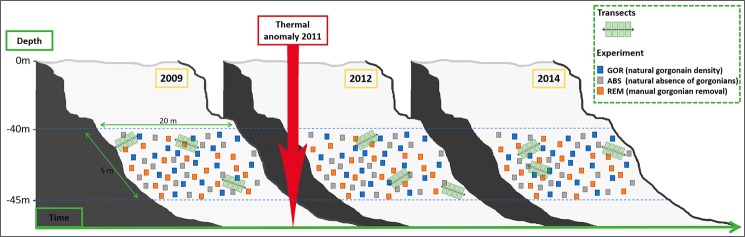
Graphical scheme of the sampling methodology for the two approaches conducted at the study site: (i) the observational study to evaluate the effects of the thermal anomaly on the whole community, and (ii) the experimental study to test the role of the *P. clavata* as a structural species. Thermal anomaly is also represented in the scheme to graphically represent the entire study context.

In this study, we take advantage of a five-year (2009–2014) monitoring study on a coralligenous assemblage unexpectedly affected by an episode of anomalous high temperatures in the Archipelago of Cabrera National Park (2011; Fig. 1). This monitoring, was combined with an experimental study, at the same location (Fig. 2), manipulating the presence of the red gorgonian *P. clavata*, to assess (1) the effects of the thermal anomaly at the community level, (2) the structural role of *P. clavata* in coralligenous habitats and (3) how the presence or absence of *P. clavata* may influence the resistance of the assemblage to the effects of a thermal anomaly and to the invasion by the alien alga *Caulerpa cylindracea*.

## Results

### 2009–2013 temperature and 2011 thermal anomaly

*In situ* temperature data showed that the extent of warm temperature exposure (>20 °C) at 40 m depth in 2011 (79 days) was much more prolonged (228%) than the mean value observed for the rest of the years (35 ± 16 days; MEAN ± SD; Fig. 1F). It was noticeable, that the exposure to the 24 °C lethal threshold in 2011 (17 days) represented a 2187% increase with respect to the mean value observed the other 4 years (0,8 days) highlighting a strong positive thermal anomaly in 2011 (Fig. 1F).

### Monitoring study to evaluate the effects of a thermal anomaly on coralligenous assemblages

A total of 72 taxa were identified across years at several taxonomic levels, 7 phyla, 20 genera and 45 species. From them, 17 taxa were macroalgae, 9 anthozoans, 2 hydrozoans, 14 bryozoans, 1 polychaete, 1 foraminifera, 21 sponges and 7 tunicates. A total of 59 species were identified on the assemblage on 2009, 53 on 2012 and 31 on 2014 (Supplementary Table S1).

**Figure 3:**
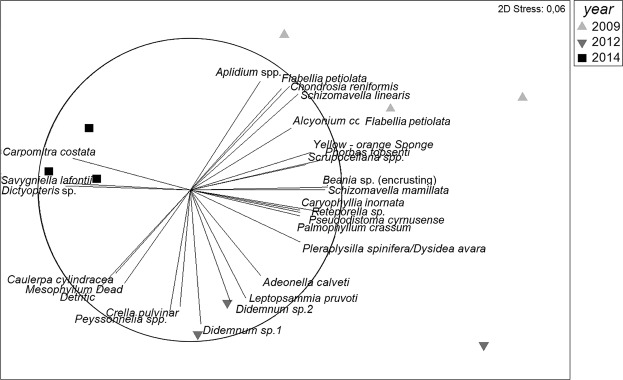
Non-metric multidimensional scaling (MDS) ordination plot of coralligenous species structure and composition over years (2009, 2012 and 2014). Distances in ordination represent differences in assemblage composition, and the overlay vectors represent the most correlated species (>0.70).

The species composition changed significantly with time (Fig. 3; p < 0.01, Supplementary Table S2). The main species contributing to the coralligenous assemblages were the encrusting algae *Mesophyllum* sp., *Peyssonnelia* spp. and *Palmophyllum crassum*; the sponges *Phorbas topsenti*, *Axinella damicornis* and *Crambe crambe*; the bryozoans *Scrupocellaria* spp. and *Schizomavella mamillata*; the tunicate *Pseudosistoma cyrnusense;* and two arbitrarily fixed categories: “turf” (consisting of small invertebrates and algae) and detritus (SIMPER analysis; Supplementary Table S3A). However, abundance of most species increased or decreased after the thermal anomaly (Supplementary Table S3B). For instance, the encrusting algae *Peyssonnelia* spp. increased (23%) its abundance while *Mesophyllum* sp. decreased (56%). The most representative sponge species (*A. damicornis* and *C. crambe*, 56% and 63% respectively), the tunicate *P. cyrnusense* (100%), the bryozoan *S. mamillata* (72%), and the cnidarian *Alcyonium acaule* (100%) decreased their abundance after the thermal anomaly, whereas “turf” and “detritus” categories increased during the last study years (100% and 127% respectively; Supplementary Table S3B). Finally, erect algae such as *Dictyopteris* sp. and *Carpomitra costata*, and bryozoans such as *Savignyella lafontii* increased (535%, 1733% and 1955% respectively) their abundance the last monitoring year (S3. B).

**Figure 4:**
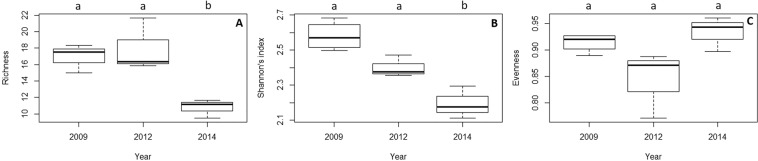
Box plot of the temporal variation of (**A**) species richness, (**B**) Shannon’s diversity index and (**C**) evenness. The median values (bold horizontal line), the interquartile distances (the box) and the extreme values, which are non-outliers (whiskers), are indicated in the plot. Significant differences between years (p-values of Tukey test with 95% confidence intervals) are indicated above boxes with letters.

In terms of diversity, the assemblage remained stable before and just after the thermal anomaly (Fig. 4; p > 0.05; Supplementary Table S4). However, species richness and Shannon’s index decreased significantly in 2014, three years after the thermal anomaly (Fig. 4; p < 0.05, Supplementary Table S4).

### Manipulative field experiment to assess the structural role of *P. clavata* and the resistance of the understory assemblage to the impacts of the thermal anomaly and invasive species

Species composition and abundances significantly changed among treatments and through time in the manipulative experiment, whereas the interaction between treatment and time was not significant (Table 1).

**Table 1:** Details of two-way PERMANOVA test (with Year, three levels and Treatment, three levels as fixed factors) for the coralligenous assemblage species abundance and composition.

	Factor	Df	SS	MS	Pseudo-F	P (perm)	P (MC)
Abundance	Treatment	2	14216	7108	8.90	***	***
	Year	2	25934	12967	16.24	***	***
	Tr × Yr	4	3539	884.76	1.11	0.29	0.29
	Qu (Tr × Yr)	167	1.33E + 05	798.98	1.44	0.24	0.27
	Residual	1	553.87	553.87			
	Total	176	1.78E + 05				
Presence/Absecence	Treatment	2	12724	6362	7.97	***	***
	Year	2	28471	14236	17.84	***	***
	Tr × Yr	4	3408.6	852.15	1.07	0.37	0.37
	Qu (Tr × Yr)	167	1.33E + 05	798.52	1.44	0.28	0.30
	Residual	1	555.56	555.56			
	Total	176	1.79E + 05				

Analysis based on Bray-Curtis similarities for Abundance and Presence/Absence species data. P-values < 0.001 are represented by ***, <0.01 by ** and <0.05 by*.

**Figure 5:**
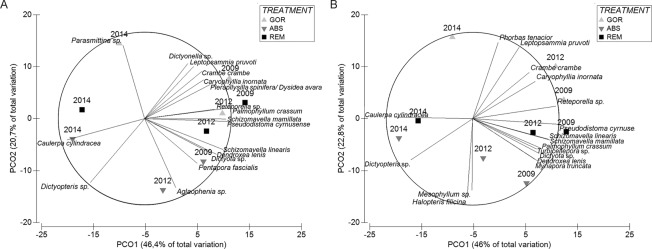
Principal coordinate analysis of (**A**) abundance species data and (**B**) presence-absence species data showing the shift in the community for the different treatments. Distances in ordination represent differences in assemblage composition, and the overlay vectors represent the most correlated species (>0.80). In the legend, GOR indicates non-manipulated plots with a natural gorgonian density, ABS non-manipulated plots where gorgonians were naturally absent and REM manipulated plots from which gorgonians were totally removed.

Principal Coordinate Ordination analysis (PCOs) shows shifts in the understory during the experiment (Fig. 5) both if we take into account species presence/absence or the abundances. The first axis in both PCOs is associated with time, being 2009 and 2012 (just after the thermal anomaly) similar among them, and different from 2014. SIMPER analysis (Supplementary Table S5) reveals that the significant changes through time were primarily related to a decrease in abundance of several erect invertebrates such as the tunicates *P. cyrnusense* and *Aplidium* sp., cnidarians *A. acaule* and *Leptopsammia pruvoti*, bryozoan *S. linearis* and some sponges (*A. damicornis* and *C. crambe*), as well as to the increase in abundance of some erect algae such as *C. cylindracea* and *Dictyopteris* sp.

The second axis of the PCO is strongly correlated with the treatment factor showing that the understory assemblage is affected by gorgonian presence. PERMANOVA pairwise test (Table 2) shows that the abundance and the presence/absence of understory species in 2009 were similar in GOR and REM plots (p > 0.05), when REM still had a natural canopy of gorgonians, while ABS plots (naturally without gorgonians) differed significantly from GOR and REM plots (p < 0.05).

**Table 2:** Details of two-way PERMANOVA pairwise test (with Year, three levels and Treatment, three levels as fixed factors) for the coralligenous assemblage species abundance and composition, Analysis based on Bray-Curtis similarities for Coverage and Presence/Absence species data.

Year	Treatment	Abundance			Presence/Absecence		
		T	P (perm)	P(MC)	T	P (perm)	P(MC)
2009	GOR,ABS	2.22	***	***	2.02	***	**
	GOR, REM	1.21	0.13	0.15	1.09	0.29	0.30
	ABS, REM	1.81	**	**	1.85	**	**
2012	GOR,ABS	2.32	***	***	2.10	***	**
	GOR, REM	2.18	***	***	2.02	***	**
	ABS, REM	1.90	***	**	1.94	***	**
2014	GOR,ABS	2.26	***	**	2.32	***	**
	GOR, REM	1	**	**	1.85	*	*
	ABS, REM	1.33	0.09	0.11	1.01	0.39	0.52

Where p-values < 0.001 are represented by ***, <0.01 by ** and <0.05 by*. In the table, GOR indicates non-manipulated plots with a natural gorgonian density, ABS non-manipulated plots where gorgonians were naturally absent and REM manipulated plots from which gorgonians were totally removed.

GOR and REM plots were characterized by a major contribution of the alga *P. crassum*, the sponges *C. crambe* and *Pleraplysilla spinifera*, the tunicate *P. cyrnusense*, the anthozoans *L. pruvoti* and *A. acaule*, and the bryozoans *S. linearis* and *Reteporella* sp. In contrast, the algae *Peyssonnelia* spp.*, Halopteris filicina*, *Dictyopteris* sp. and *Dictyota* sp., the sponge *A. damicornis*, and “detritus” category contributed more to the coverage of ABS plots (Supplementary Table S6).

After the thermal anomaly (2012) all treatments were significantly different from each other, while at the end of the experiment (2014) the understory of REM and ABS plots (both now without gorgonians) became similar (p > 0.05; Table 2) and significantly different (p < 0.05) from GOR plots (with gorgonians). In 2014, all treatment plots were characterized by a high abundance of turf, the alga *Mesophyllum* sp. and the sponge *P. topsenti*. However, GOR plots displayed higher abundances of macroinvertebrates such as *L. pruvoti, C. crambe, P. cyrnusense* and *S. mamillata*, and lower abundances of algae *C. cylindracea*, *Dictyopteris* sp., *H. filicina, Peyssonnelia* spp., and sponge *A. damicornis*, than the REM and ABS plots (Supplementary Table S7). Therefore, in general, the presence of gorgonians (GOR) favored macroinvertebrates in contrast to algae.

**Figure 6:**
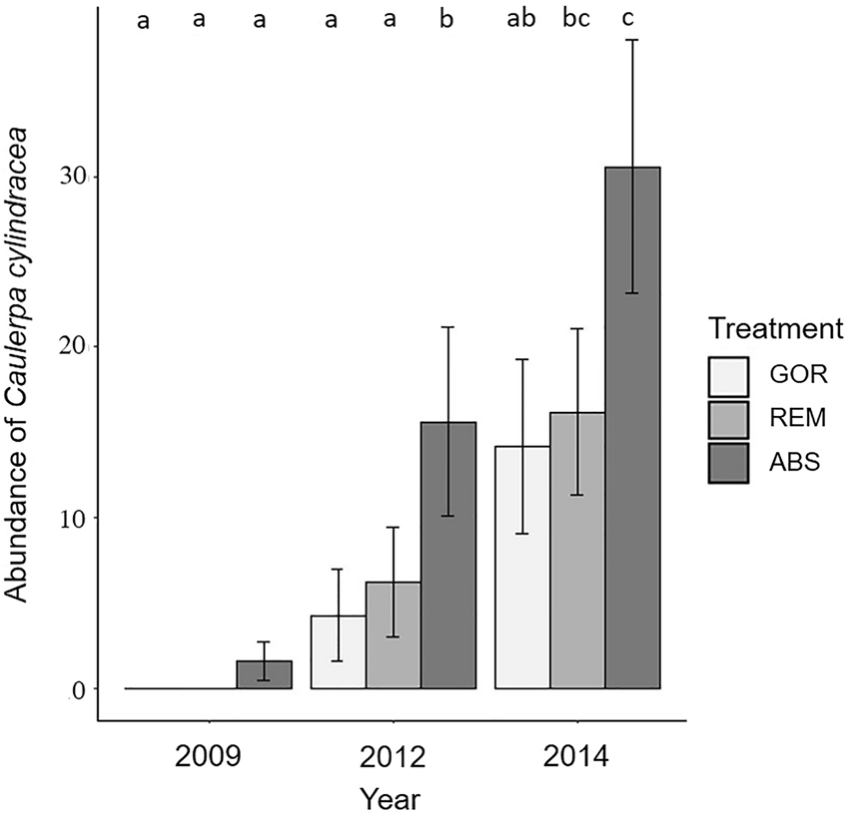
Representation of the mean values of the percentage abundance of *C. cylindracea* for each treatment and year with standard error bars. Significant differences between factors (p-values of pairwise comparisons with 95% confidence intervals) are indicated above bars with letters. In the legend, GOR indicates non-manipulated plots with a natural gorgonian density, ABS non-manipulated plots where gorgonians were naturally absent and REM manipulated plots from which gorgonians were totally removed.

Time and treatment had a significant effect on *C. cylindracea* abundance (Supplementary Table S8). However, the significant interaction between both factors indicates that the *C. cylindracea* increase with time was dependent on the presence of gorgonians. All treatments exhibited a pattern of *C. cylindracea* increase over time but its abundance on ABS plots was always higher, increasing from 2009 to 2012 (p < 0.05; Fig. 6) and from 2012 to 2014 (p < 0.05; Fig. 6). After the removal of gorgonians (REM treatment) *C. cylindracea* also significantly increased from 2012 to 2014 (p < 0.05; Fig. 6). In contrast, there was not a significant increase of *C. cylindracea* in GOR plots between 2012 and 2014 (p > 0.05; Fig. 6).

**Figure 7:**
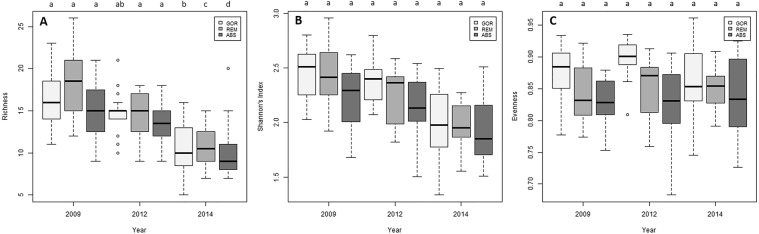
Box plots of the temporal variation of (**A**) species richness, (**B**) Shannon’s index diversity and (**C**) evenness for each treatment. The median values (bold horizontal line, the interquartile distances (the box), the extreme values, which are non-outliers (whiskers), and the outliers (spots) are indicated in the plot. Significant differences between factors (Year and Treatment, p-values of pairwise comparisons with 95% confidence intervals) are indicated above boxes with letters. In the legend, GOR indicates non-manipulated plots with a natural gorgonian density, ABS non-manipulated plots where gorgonians were naturally absent and REM manipulated plots from which gorgonians were totally removed.

Species richness experimented a significant decrease along the experiment in the three treatments (Fig. 7A; Supplementary Table S9), but no variation with time was detected in the Shannon’s diversity and evenness indices in any treatment (Fig. 7B,C; Supplementary Table S9).

## Discussion

*Paramuricea clavata* mortalities have been exclusively attributed to temperature anomalies and diver frequentation^33^. Considering the low anthropogenic pressures and the lack of diving activities in the study site, we pose that temperature anomaly is the most probable cause that explains *P. clavata* mortalities at the study site. In fact, temperature during summer 2011 was exceptionally higher (Fig. 1), exceeding 24 °C during 17 days at the study depth, which is enough to trigger a mortality event in *P.clavata*^21,24,39–42^. In fact, Linares *et al*. (2017)^43^ report a severe affectation of this temperature anomaly on the population of *P. clavata* at the study site. The understory assemblage also showed effects of this anomaly. However, we show that the delayed (2.5 years) effects were more severe and had far-reaching implications than the immediate effects, which highlights the relevance of examining the effects of climatic driven events in benthic assemblages at different time scales^27^. The assemblage shifted from being dominated by erect invertebrates and encrusting sponges to be dominated by erect algae and turf-forming species. Several macroinvertebrates and coralline algae usually dwelling in the understory of *P. clavata* have been reported to be affected by thermal anomalies^21,24,44–49^ and thus, the changes observed in the whole assemblage were expected. Although the fine-tuning mechanisms behind the impacts of a thermal anomaly are difficult to ascertain, it has been reported that many organisms inhabiting coralligenous assemblages suffer from partial mortality or physiological stress^41,50–52^, which may led to their total mortality in subsequent years^26^. The final result has been a simplification in terms of biodiversity and structure, with losers (e.g. species that are sensitive to thermal stress such as macroinvertebrates) and winners (species that can outcompete the sensitive species and are resistant to thermal anomalies such as turf-forming species).

Moreover, calcareous matrix coming from dead organisms led to an increase of detritus after the thermal anomaly. Detritus accumulate as sediment above the substrate which has a direct deleterious effects on coralligenous outcrops by inhibiting recruitment and promoting burial and scouring of macroinvertebrates^53–56^. In turn, heavy sedimentation also facilitates turf growth^55,57^, which inhibits the recruitment and increases the mortality of juvenile gorgonians and corals^23,58–60^ as well as decreases the reproductive output of several sponges^61^. The higher abundance of turf-forming species is also probably behind the increase of *C. cylindracea*, as this species is facilitated by the abundance of turf-forming species^62^. Cumulative impacts of warming, sedimentation, turf algae overgrowth and invasions may drive a snow-ball effect, speeding up the mid-term shift in the understory assemblage.

The significant effects of the presence/absence of *P. clavata* on the understory species clearly reveals the role of *P. clavata* as a habitat forming species, favoring diversity and structure of the understory. Promoting invertebrate settlement and preventing turf growth appear not to be limited to gorgonian forests^63–68^, as it has also been reported for other habitat-forming species such as kelps, which facilitate settlement of sponges and coralline algae and inhibit the presence of turf-forming algae^69,70^.

*Paramuricea clavata* has also a mitigation effect of warming temperatures over invertebrates thriving on the understory, although the influence of the thermal anomaly is also noteworthy. In fact, detritus and turf even increased in the presence of *Paramuricea*, what may compromise the survival of the remaining macroinvertebrates in the long-term.

Finally, although *P. clavata* does not prevent the establishment of *C. cylindracea*, it is able to delay its proliferation and spread, probably because complexity of substrata (enhanced by gorgonians presence) is a key factor to limit colonization and spread of *C. cylindracea* in Mediterranean habitats^62,71^. Thus, we hypothesize that extreme climatic events may be indirectly promoting the invasion of *C. cylindracea* in coralligenous assemblages by directly causing the mass mortality of structural native species and by indirectly increasing the abundance of turf-forming species. Moreover, the interaction of various stressors, such as warming and invasive species, may cause additive effects that end up in catastrophic ecosystem changes^15,72^.

Ongoing environmental changes are predicted to increase the frequency and intensity of extreme climatic events^73,74^. Here, we show how an extreme warming event can replace a structurally complex habitat dominated by long-lived gorgonians by a simplified habitat dominated by turf-forming species, with a generalized vulnerability to be colonized by invasive species. Thus, we bring evidence of the synergic and additive effects of global (e.g. warming) and local stressors (e.g. invasions) that are affecting ecosystems around the world^15,75^. Bearing in mind that climatic models predict that the Mediterranean Sea will be one of the regions most affected by warming and extreme climatic events^76^, our study is especially relevant as it shows a degradation of an entire Mediterranean assemblage to levels never reported before. Whether this process is generally applicable to other assemblages or ecosystems is still unknown, but if these shifts at the ecosystem level regularly occur, the impoverishment of the ecological quality of Mediterranean benthic assemblages, driven by climate change, can happen at stronger and faster rates than assumed.

## Methods

### Study site

The study site is located in Cabrera National Park (Balearic Islands, western Mediterranean), a remote access area, far away from the coast. In Cabrera NP dissolved inorganic nutrients (DIN), Chla and trace metals are characteristic of oligotrophic Mediterranean waters^77,78^. Ecological Quality Ratio for all bioindicators (macroalgae, *Posidonia oceanica*, phytoplankton) and physicochemical parameters evaluated following European Water Framework Directive (WFD, 2000/60/EC) are always above 0.9 units, being 1 the value corresponding to the highest ecological status^79,80^. In Cabrera NP there is no potential sources of pollution (i.e. industrial, agricultural, dumping, mining or dredging), and human density is 0.9 persons·km^−2^. There are no rivers or industry. The water is extraordinarily clear: Secchi Disk depth oscillates between 24 m (February) and 38 m (August), whereas the mean annual light extinction coefficient (k) is 0.063 m^−1^ ^81^. Water temperatures are characteristic of those reported for the Balearic basin, with minimal temperatures around 14 °C and surface temperatures reaching values of 27 °C^77,81^. Salinity is almost constant in the Mediterranean Sea and ranges in the area from 37.5% to 38.1%^77^. No significant acidification has been reported in the area.

The studied assemblage was located on a vertical rocky wall between 40 and 45 m depth, facing southeast at the Imperial Islet (39° 07′34″N; 2°57′29″E)^82^, where diving is completely forbidden, except for scientific purposes.This area suffered the effects of positive thermal anomalies, which mainly affected sublittoral assemblages from 5 to 45 m depth^28,43^. These anomalous high-temperature conditions were identified as the primary causal factor for the mass mortalities of two abundant invertebrates, the sponge *Sarcotragus fasciculatus* and the gorgonian *P. clavata*^28,43^. Specifically, from 2009 to 2014, two stress factors affected the studied assemblage: (i) a thermal anomaly in 2011, and (ii) the invasion of *C. cylindracea*, which was first detected in the area in 2008^83,84^.

### Monitoring thermal environment

The thermal environment damaged the gorgonian population situated between 37 and 45 m depth affecting 90% of the colonies^43^. The thermal anomaly was studied by deploying *in situ* high-resolution (hourly records, ±0.21 °C accuracy) temperature recorders (HOBO Water Temp Pro v2). Temperature loggers were placed at the study site in intervals of 5 m between 5 to 67 m depth and were changed every two years. We used the number of days that the community was exposed to ≥20 °C as *a proxy* of the extent of warm temperature exposure and, the number of days that the community was exposed to 24 °C as a *proxy* of the extent of lethal threshold exposure^40–42^ (Fig. 1).

### Monitoring study to evaluate the effects of a thermal anomaly on coralligenous assemblages

Photo-quadrats along transects were used to study the changes in species composition and abundances from 2009 to 2014 (Fig. 2). Samplings were performed in May 2009, 2012 and 2014. Periodicity was selected according to the low dynamics and stability of these assemblages (with no temporal changes in biodiversity patterns over more than 5 years^38^) added to the low accessibility to the study site (at 40–45 m depth in a remote area). Each year, three (2 m-long) transects were randomly deployed between 40 and 45 m depth. Eight photographs of 25 × 25 cm of the understory community were taken per transect as described by Kipson *et al*. (2011)^85^. The sampling unit selected (5000 cm^2^ per transect) was in accordance to the minimal sampling area proposed for coralligenous assemblages dominated by the gorgonian *P. clavata*^57^. Pictures were obtained with a Nikon D70S digital SLR camera fitted with a Nikon 20 mm DX lens (3000 * 2000-pixel resolution) and housed in a Subal D70S housing. Lighting was achieved using two electronic strobes fitted with diffusers. Sessile macro-taxa were identified in each picture to the lowest possible taxonomic level, and the abundance of each taxon was measured. Image analysis with Adobe Photoshop software was used to estimate the abundance for each species by means of a superposed reticulum (of 25 × 25 cm, divided in 25 sub-quadrats), and the number of sub-quadrats in which each species appeared was recorded and used as unit of abundance^86^. Species richness and Shannon’s diversity index were calculated from the data acquired.

### Manipulative field experiment to assess the structural role of *P. clavata* and the resistance of the understory assemblage to the impacts of the thermal anomaly and invasive species

The manipulative experiment was performed at the same coralligenous assemblage than the monitoring study. To assess the role of *P. clavata* as a structural species and to determine whether its presence can modify the response of the whole assemblage to thermal anomalies, a field experiment manipulating gorgonians presence was conducted from May 2009 to May 2014 at a depth between 40 and 45 m (at the same site than the monitoring; Fig. 2). Three treatments of 20 randomly distributed 25 × 25 cm plots each were set up: a) non-manipulated plots with a natural gorgonian density [approximately 20 colonies/m^2^ ^42^; GOR], b) non-manipulated plots where gorgonians were naturally absent (ABS), and c) manipulated plots from which gorgonians were totally removed (REM). To minimize possible variation due to local environmental factors, the different replicates of each treatment were located interspaced in an approximately 5 × 20 m area (Fig. 2). To assess species composition and abundances in the different treatments plots over time (before and after the occurrence of the thermal anomaly in 2011), photo sampling was again performed in May 2009, 2012 and 2014. Pictures were analyzed as in the monitoring study. Species richness and Shannon’s diversity index were calculated from the data acquired for each treatment and sampling date.

### Statistical analysis

#### Monitoring study

Shifts in species composition and abundances over time at the assemblage level, were analyzed by non-metric multidimensional scaling (MDS) based on Bray-Curtis similarity. Data was previously fourth root transformed to mitigate the effects of the most abundant species^87^ and the most correlated species with the ordination axes (Pearson’s correlation > 0.7) were represented as overlaying vectors. One-way multivariate PERMANOVA (non-parametric analysis of variance) also based on the Bray-Curtis similarity on fourth root transformed data, with year as a fixed factor (3 levels 2009, 2012 and 2014), was performed to analyze species abundance variation through time. Pairwise comparisons were performed to test for differences between years^88^. Due to the low possible number of permutations (<999), P-values provided by Monte Carlo test were used in preference^89^. Moreover, SIMPER analyses for the years 2009 and 2014 were performed to identify the species that contributed the most to the assemblage change after the thermal anomaly.

Species richness, Shannon’s diversity and evenness indices were the descriptors used to analyze temporal variations in the target assemblage. One-way ANOVA with year as a fixed factor (three levels: 2009, 2012 and 2014) was performed for each descriptor. Data were previously tested for normal distribution and homogeneity of variances using Shapiro-Wilk normality test and Bartlett’s test respectively (p-values > 0.05). For those variables proving significant in the ANOVA, differences between concrete pairs of years were analyzed by posterior Tukey’s test.

#### Manipulative experiment

To assess the role of *P. clavata* as a structural species, we analyzed the effects of its loss on the assemblages subjected to the thermal anomaly. Assemblage composition and structure were analyzed both on species abundance (data previously fourth root transformed) and on presence-absence data with a three-way multivariate PERMANOVA based on the Bray-Curtis similarity, performed with 9999 unrestricted random permutations^88^. The sampling design included three factors: treatment (as a fixed factor with three levels: GOR, ABS and REM), year (fixed factor with three levels: 2009, 2012 and 2014) and plot (as a random factor, nested in treatment and year). Subsequent pairwise comparisons for all combinations of treatment and year were carried out. To show the temporal trends of the different treatments, a Principal Coordinate Analysis (PCO) based on Bray-Curtis similarity was performed and vectors representing the most correlated species with the ordination axes (Pearson’s correlation > 0.8) were overlaid. *P. Paramuricea clavata* data was excluded from the analysis, because its presence was manipulated.

Finally, variations in the abundance of the alien alga *C. cylindracea*, the species richness, Shannon’s and evenness indices of the assemblage, were analyzed along time and between treatments. Generalized linear mixed models (GLMM) with three factors (Year and Treatment as fixed factors and Plot as random) were used, as data failed to comply normality assumptions (Saphiro-Wilk test p-value < 0.05), and GLMMs are suitable for non-normal data^90^ and repeated measures over time^91^. All analyses were computed using the program Primer 6 + PERMANOVA^92,93^ and R software^94^ with Vegan^95^ and lme4^96^ packages.

## Supplementary information

Supplementary information

## Data Availability

Data Availability Statement: The authors agree to archive all data supporting the results of this paper in an appropriate public archive, if it is accepted for publication.
