# Supplementary material for: Biodiversity loss in a Mediterranean ecosystem due to an extreme warming event unveils the role of an engineering gorgonian species
Source: Sci Rep. 2019 Apr 11;9:5911. doi: 10.1038/s41598-019-41929-0 (PMC6459914; doi:10.1038/s41598-019-41929-0)
Supplement: Supplementary file 1 — Supplementary information [file 41598_2019_41929_MOESM1_ESM.pdf]

"Biodiversity loss in a Mediterranean ecosystem due to an extreme warming event unveils the role of an engineering gorgonian species"

Jana Verdura[1,2], Cristina Linares[3], Enric Ballesteros[2], Rafel Coma[2], María J. Uriz[2], Nathaniel Bensoussan[4,5], Emma Cebrian[1,2*].

1. Institut d'Ecologia Aquàtica, Facultat de Ciències, Universitat de Girona, Campus Montilivi, 17071 Girona, Spain

2. Centre d'Estudis Avançats de Blanes-CSIC, Accés Cala Sant Francesc 14, 17300 Blanes, Girona, Spain

3. Department of Evolutionary Biology, Ecology and Environmental Sciences, Institut de Recerca de la Biodiversitat (IRBIO), University of Barcelona, Av. Diagonal 643, 08028 Barcelona, Spain

972 33 61 01

4. Institut Ciències del Mar, CSIC, Barcelona, Spain

5. Aix Marseille University, Université de Toulon, CNRS, IRD, MIO, Marseille, France

*Corresponding author: e-mail: emma.cebrian@udg.edu, emma@ceab.csic.es. Tel: +34 972419823, +34 972 33 61 01

Supplementary Information

Supplementary Table S1. List of identified taxa in the different years (+ means presence, - means absence).

Species	2009	2012	2014
Bryopsis plumosa	+	-	-
Carpomitra costata	-	-	+
Caulerpa cylindracea	+	+	+
Corallina elongata	+	-	-
Dictyopteris sp.	+	+	+
Dictyota sp.	+	+	+
Flabellia petiolata	+	-	-
Halimeda tuna	+	+	+
Halopteris filicina	+	+	+
Kallymenia sp.	+	-	-
Mesophyllum sp.	+	+	+
Palmophyllum crassum	+	+	+
Peyssonnelia spp.	+	+	+
Rhodymenia sp.?	+	+	+
Valonia sp.	+	-	-
Phyllariopsis brevipes	-	-	+
Zanardinia typus	+	+	+
Axinella damicornis	+	+	+
Chondrilla nucula	-	+	-
Chondrosia reniformis	+	-	-
Clathrina sp. (white)	-	+	-
Cliona viridis	+	+	+
Corticium sp.	+	-	-
Crambe crambe	+	+	+
Crella pulvinar	+	+	+
Dendroxea lenis	-	+	+
Dictyonella sp.	-	+	+
Haliclona mucosa	+	+	-
Haliclona mediterranea	+	-	-
Hemimycale columella	-	+	-
Phorbas tenacior	+	+	+
Phorbas topsenti	+	+	+
Pleraplysilla spinifera/Dysidea avara	+	+	-
Orange Sponge 1	+	+	+
Orange Sponge 2	+	-	-
Yellow - orange Sponge	+	+	-

White sponge	+	-	+
Terpios fugax	+	-	-
Aglaophenia sp.	-	+	-
Eudendrium sp.	+	+	+
Alcyonium acaule	+	+	-
Caryophyllia inornata	+	+	-
Cerianthus membranaceus	+	-	-
Hoplangia durotrix	-	+	-
Leptopsammia pruvoti	+	+	+
Paramuricea clavata	+	+	+
Parazoanthus axinellae	+	+	-
Alcyonium coralloides	+	-	-
Polycyathus muellerae	-	+	-
Adeonella calveti	+	+	-
Beania sp. (encrusting)	+	+	-
Caberea boryi	+	+	-
Cellaria salicornioides	+	+	-
Crisia spp.	+	+	-
Myriapora truncata	+	+	+
Pentapora fascialis	+	+	-
Reteporella sp.	+	+	-
Savignyella lafontii	-	+	+
Schizomavella mamillata	+	+	+
Schizomavella linearis	+	+	-
Parasmittina sp.	-	+	+
Rhynchozoon spp.	+	+	-
Scrupocellaria spp.	+	+	+
Aplidium sp.	+	-	-
Halocynthia papillosa	+	-	-
Didemnum sp.1	-	+	-
Didemnum sp.2	+	+	-
Didemnidae sp. (white-grey)	+	-	-
Pycnoclavella communis	+	+	-
Pseudodistoma cyrnusense	+	+	-
Miniacinia miniacea	+	-	-
Salmacina dysteri	+	+	+

Supplementary Table S2. Results of one-way PERMANOVA based on Bray-Curtis similarity index performed on coralligenous assemblages of the different sampled years. P-values <0.001 are represented by ***, <0.01 by ** and <0.05 by*.

Factor	Df	*SS*	MS	Pseudo-F	P (perm)	P (MC)
Year	2	4335,3	2167,6	4,41	***	**
Residual	7	3435,6	490,8			
Total	9	7770,9				

Supplementary Table S3. Summary of SIMPER analysis for the years 2009 and 2014, listing the species by their contribution **A)** to the coralligenous assemblage per each year, and **B)** to the observed changes in the coralligenous assemblage between years. The direction of change represents the increase (+) or decrease (-) of the species abundance among years.

A) Similarities

2009

Species	Av.Abund	Av.Sim	Sim/SD	Contrib%	Cum.%
Mesophyllum sp.	1,88	6,96	13,8	10,05	10,05
Turf invertebrate	1,76	6,64	34,96	9,6	19,65
Turf algae	1,48	5,73	20,73	8,28	27,93
Phorbas topsenti	1,54	5,41	6,29	7,82	35,76
Scrupocellaria spp.	1,47	5,06	7,08	7,31	43,07
Schizomavella mamillata	1,44	4,98	8,17	7,2	50,27
Schizomavella linearis	1,05	3,68	8,01	5,31	55,59
Pseudodistoma cyrnusense	1,14	3,66	4,71	5,29	60,88
Peyssonnelia spp.	1,05	3,16	3,67	4,56	65,44
Axinella damicornis	0,84	2,66	9,87	3,84	69,28
Crambe crambe	0,89	2,43	2,02	3,51	72,79
Palmophyllum crassum	0,67	2	2,91	2,89	75,68
Alcyonium acaule	0,63	1,84	3,08	2,65	78,34
Reteporella sp.	0,41	1,23	2,17	1,77	80,11
Aplidium sp.	0,55	1,19	1,76	1,72	81,83
Pentapora fascialis	0,4	1,17	2,94	1,7	83,52
Detritus	0,52	1,17	1,37	1,7	85,22
Rhodymenia sp.?	0,56	1,12	0,88	1,62	86,84
Cliona viridis	0,35	0,89	2,43	1,29	88,13
Flabellia petiolata	0,41	0,74	0,76	1,06	89,19
Yellow - orange Sponge	0,26	0,63	0,91	0,91	90,1

2014

Turf invertebrate	1,83	9,36	10,97	11,9	11,9
Turf algae	1,66	8,57	20,75	10,88	22,78
Mesophyllum sp.	1,53	7,53	8,79	9,57	32,35
Peyssonnelia spp.	1,3	6,76	19,98	8,59	40,94
Dictyopteris sp.	1,12	5,58	30,72	7,08	48,02
Detritus	1,22	5,54	4,32	7,04	55,07
Savignyella lafontii	1,01	4,93	9,58	6,27	61,33
Phorbas topsenti	0,93	4,49	27,28	5,71	67,04
Carpomitra costata	0,94	4,22	3,04	5,37	72,41
Scrupocellaria spp.	0,94	3,47	1,74	4,41	76,82
Schizomavella mamillata	0,72	3,35	5,57	4,26	81,08
Axinella damicornis	0,71	3,03	5,41	3,85	84,93
Caulerpa cylindracea	0,49	2,24	21,54	2,84	87,78
Crambe crambe	0,48	1,6	1,33	2,03	89,8
Crella pulvinar	0,59	1,48	1,48	1,88	91,69

**B) Dissimilarities**	**Group**						
	**2009**	**2014**					
**Taxa**	**Av. Abund**	**Av. Abund**	**Av.Diss**	**Diss/SD**	**Contrib%**	**Cum.%**	**Direction of change**
Pseudodistoma cyrnusense	1,14	0	2,58	3,77	5,47	5,47	-
Schizomavella linearis	1,05	0	2,36	8,25	5,01	10,49	-
Savignyella lafontii	0	1,01	2,28	9,79	4,84	15,33	+
Carpomitra costata	0	0,94	2,14	4,02	4,54	19,86	+
Dictyopteris sp.	0,3	1,12	1,83	4,1	3,89	23,76	+
Schizomavella mamillata	1,44	0,72	1,61	2,74	3,42	27,18	-
Detritus	0,52	1,22	1,6	1,7	3,41	30,59	+
Alcyonium acaule	0,63	0	1,42	2,97	3,01	33,6	-
Phorbas topsenti	1,54	0,93	1,37	2,24	2,9	36,5	-
Scrupocellaria spp.	1,47	0,94	1,27	1,38	2,7	39,21	-
Aplidium sp.	0,55	0	1,25	1,51	2,66	41,86	-
Rhodymenia sp.?	0,56	0,07	1,2	1,44	2,54	44,4	-
Crella pulvinar	0,13	0,59	1,19	1,33	2,53	46,93	+
Crambe crambe	0,89	0,48	1,15	1,65	2,45	49,38	-
Halopteris filicina	0,44	0,29	1,05	1,13	2,24	51,62	-
Palmophyllum crassum	0,67	0,23	0,99	1,88	2,1	53,72	-
Caulerpa cylindracea	0,07	0,49	0,95	3,05	2,03	55,75	+
Reteporella sp.	0,41	0	0,94	2,6	2,01	57,76	-
Flabellia petiolata	0,41	0	0,94	1,27	2	59,76	-
Halimeda tuna	0,4	0,17	0,92	1,4	1,95	61,71	-
Pentapora fascialis	0,4	0	0,9	3,06	1,9	63,61	-
Peyssonnelia spp.	1,05	1,3	0,83	1,79	1,76	65,37	+
Leptopsammia pruvoti	0,27	0,48	0,82	1,21	1,74	67,11	+

Mesophyllum sp.	1,88	1,53	0,8	1,51	1,69	68,81	-
Bryopsis plumosa	0,33	0	0,75	0,83	1,59	70,4	-
Parazoanthus axinellae	0,32	0	0,71	1,2	1,5	73,44	-
Kallymenia sp.	0,3	0	0,68	1,31	1,45	74,89	-
Salmacina dysteri	0,34	0,25	0,67	1,24	1,41	76,3	-
Dictyota sp.	0,16	0,38	0,67	1,54	1,41	77,72	+
Cliona viridis	0,35	0,07	0,63	1,58	1,34	79,06	-
Yellow - orange Sponge	0,26	0	0,58	1,64	1,23	80,29	-
Axinella damicornis	0,84	0,71	0,57	1,13	1,22	81,51	-
Adeonella calveti	0,26	0	0,57	1,03	1,22	82,73	-
Phorbas tenacior	0,13	0,36	0,52	1	1,1	83,83	+
Caryophyllia inornata	0,23	0	0,51	0,71	1,08	84,91	-
Pycnoclavella communis	0,22	0	0,48	1,1	1,03	85,93	-
Cellaria salicornioides	0,2	0	0,46	0,83	0,98	86,91	-
Alcyonium coralloides	0,21	0	0,46	1,28	0,98	87,89	-
Turf algae	1,48	1,66	0,42	2,47	0,9	88,79	+
Pleraplysilla spinifera/Dysidea avara	0,17	0	0,37	0,55	0,79	89,57	-
Beania sp. (encrusting)	0,16	0	0,36	0,79	0,76	90,33	-

Supplementary Table S4) P-values of the one-way ANOVAs (factor year) of the richness, evenness and Shannon's Index of the coralligenous assemblage. P-values <0.001 are represented by ***, <0.01 by ** and <0.05 by*.

Variable	Factor	Df	Mean Sq	p- value
Richness	Year	2	47,41	**
	Residuals	7	4,25	
Shannon's index	Year	2	0,128	**
	Residuals	7	0,006	
Evenness	Year	2	0,007	0,05
	Residuals	7	0,0003	

Supplementary Table S5. Summary of SIMPER analysis for the YEAR factor of the experimental plots. The direction of change represents the increase (+) or decrease (-) of the species abundance between 2009 and 2014 (before and after the thermal anomaly). The factor TREATMENT was not considered to perform this analysis.

	Group						
	2009	2014					
**Taxa**	**Av. Abund**	**Av. Abund**	**Av.Diss**	**Diss/SD**	**Contrib%**	**Cum.%**	**Direction of change**
Caulerpa cylindracea	0,05	0,92	2,11	1,05	4,59	4,59	+
Pseudodistoma cyrnusense	1,1	0,41	1,94	1,41	4,21	8,81	-
Schizomavella linearis	0,77	0,05	1,73	1,31	3,76	12,57	-
Alcyonium acaule	0,76	0,02	1,67	1,12	3,62	16,19	-
Detritus	0,75	0,76	1,65	1,18	3,59	19,77	+
Crambe crambe	1,06	0,72	1,65	1,15	3,59	23,36	-
Dictyopteris sp.	0,44	0,82	1,63	1,08	3,54	26,9	+
Leptopsammia pruvoti	0,75	0,63	1,55	1,07	3,38	30,27	-
Halopteris filicina	0,76	0,55	1,53	1,04	3,32	33,6	-
Palmophyllum crassum	0,78	0,44	1,53	1,2	3,32	36,91	-
Reteporella sp.	0,67	0,08	1,51	1,15	3,27	40,19	-
Schizomavella mamillata	1,4	0,94	1,35	1,08	2,94	43,12	-
Axinella damicornis	0,79	0,66	1,35	1,09	2,93	46,05	-
Aplidium sp.1	0,59	0,05	1,29	1,04	2,8	48,85	-
Peyssonnelia spp.	1,3	1,25	1,22	0,96	2,65	51,51	-
Pleraplysilla spinifera/Dysidea avara	0,49	0,12	1,12	0,91	2,43	53,94	-
Raspaciona aculeata	0,36	0,34	1,1	0,89	2,39	56,33	-
Flabellia petiolata	0,3	0,29	1,04	0,77	2,25	58,58	-
Dictyota sp.	0,4	0,08	0,97	0,7	2,11	60,7	-
Zanardinia typus	0,23	0,27	0,87	0,67	1,9	62,59	+
Phorbas topsenti	1,59	1,38	0,87	0,87	1,88	64,48	-
Phorbas tenacior	0,21	0,25	0,85	0,62	1,84	66,32	+
Cliona viridis	0,28	0,15	0,84	0,65	1,83	68,15	-
Caryophyllia inornata	0,31	0,12	0,83	0,65	1,8	69,95	-
Dictyonella sp.	0,29	0,14	0,79	0,66	1,71	71,66	-
Orange Sponge 3	0,19	0,21	0,75	0,66	1,62	73,28	+
Turf algae	1,73	1,8	0,74	0,89	1,6	74,89	+
Crella pulvinar	0,27	0,12	0,74	0,63	1,6	76,49	-
Pentapora fascialis	0,32	0	0,7	0,59	1,52	78,01	-
Mesophyllum sp.	1,92	1,75	0,7	0,89	1,52	79,52	-
Alcyonium coralloides	0,29	0	0,65	0,56	1,42	80,94	-
Carpomitra costata	0,04	0,23	0,64	0,46	1,39	82,33	+
Halimeda tuna	0,22	0,09	0,63	0,54	1,38	83,71	-
Parasmittina sp.	0,09	0,22	0,58	0,5	1,27	84,98	+

Didemnum sp.1	0,18	0,12	0,55	0,56	1,19	86,17	-
Dendroxea lenis	0,22	0,02	0,5	0,51	1,08	87,24	-
Parazoanthus axinellae	0,19	0,05	0,48	0,52	1,04	88,29	-
Turf invertebrate	1,89	1,96	0,47	1,23	1,01	89,3	+
*Myriapora truncat*a	0,19	0,03	0,45	0,48	0,98	90,28	-

Supplementary Table S6. Summary of SIMPER analysis for the year 2009. The percentage of contribution of each species within the overall assemblage is shown for the plots harbouring gorgonian forests (GOR and REM) and assemblages without gorgonians (ABS). The direction of change represents the increase (+) or decrease (-) of the species abundance between GOR and REM VS ABS

	Group 2009						
	GOR+REM	ABS					
**Taxa**	**Av. Abund**	**Av. Abund**	**Av.Diss**	**Diss/SD**	**Contrib%**	**Cum.%**	**Direction of change**
Crambe crambe	1,31	0,55	1,83	1,33	4,46	4,46	-
Detritus	0,59	1,05	1,75	1,28	4,25	8,71	+
Halopteris filicina	0,58	1,11	1,61	1,24	3,92	12,63	+
Leptopsammia pruvoti	0,91	0,43	1,54	1,24	3,74	16,37	-
Alcyonium acaule	0,77	0,73	1,43	1,2	3,49	19,86	-
Dictyopteris sp.	0,33	0,68	1,37	1,1	3,33	23,19	+
Pleraplysilla spinifera/Dysidea avara	0,67	0,15	1,26	1,14	3,07	26,26	-
Reteporella sp.	0,75	0,52	1,25	1,17	3,03	29,29	-
Axinella damicornis	0,72	0,92	1,23	1,14	3	32,29	+
Schizomavella linearis	0,8	0,73	1,21	1,1	2,93	35,23	-
Palmophyllum crassum	0,79	0,76	1,2	1,12	2,93	38,16	-
Pseudodistoma cyrnusense	1,19	0,92	1,19	1,05	2,9	41,06	-
Dictyota sp.	0,35	0,48	1,18	0,91	2,87	43,93	+
Aplidium sp.1	0,57	0,61	1,17	1,09	2,85	46,77	+
Pentapora fascialis	0,24	0,46	1,03	0,9	2,51	49,28	+
Cliona viridis	0,2	0,45	1,01	0,83	2,45	51,73	+
Raspaciona aculeata	0,35	0,38	0,98	0,92	2,38	54,11	+
Peyssonnelia spp.	1,31	1,26	0,94	1	2,28	56,4	-
Caryophyllia inornata	0,44	0,06	0,93	0,78	2,26	58,66	-
Flabellia petiolata	0,28	0,34	0,92	0,8	2,23	60,88	+
Halimeda tuna	0,13	0,4	0,91	0,79	2,21	63,1	+
Alcyonium coralloides	0,34	0,2	0,86	0,76	2,1	65,2	-
Dictyonella sp.	0,33	0,22	0,83	0,76	2,01	67,21	-
Crella pulvinar	0,32	0,19	0,8	0,73	1,94	69,15	-
Zanardinia typus	0,21	0,27	0,79	0,71	1,92	71,08	+
Schizomavella mamillata	1,4	1,39	0,72	0,83	1,75	72,83	-
Dendroxea lenis	0,25	0,18	0,67	0,67	1,64	74,47	-
Turbicellepora sp.	0,17	0,23	0,64	0,64	1,57	76,04	+
Phorbas tenacior	0,32	0	0,62	0,54	1,51	77,54	-
Turf algae	1,66	1,86	0,61	0,81	1,48	79,02	+
Parazoanthus axinellae	0,18	0,2	0,61	0,66	1,47	80,49	+
Myriapora truncata	0,19	0,18	0,59	0,61	1,44	81,93	-
Mesophyllum sp.	1,84	2,06	0,58	0,75	1,4	83,34	+
Orange Sponge 3	0,26	0,05	0,57	0,61	1,39	84,73	-
Aglaophenia sp.	0,11	0,22	0,57	0,58	1,38	86,11	+

Adeonella calveti	0,18	0,16	0,54	0,61	1,31	87,41	-
Didemnum sp.1	0,25	0,05	0,51	0,57	1,25	88,66	-
Phorbas topsenti	1,58	1,61	0,49	0,82	1,2	89,86	+
Hoplangia durotrix	0,22	0	0,44	0,45	1,07	90,93	-

Supplementary Table S7. Summary of SIMPER analysis for the year 2014. The percentage of contribution of each species within the overall assemblage is shown for the plots GOR versus REM and ABS. The direction of change represents the increase (+) or decrease (-) of the species of GOR treatments vs REM and ABS.

	Group 2014						
	GOR	ABS+REM					
**Taxa**	**Av. Abund**	**Av. Abund**	**Av. Diss**	**Diss/SD**	**Contrib%**	**Cum.%**	**Direction of change**
Leptopsammia pruvoti	1,09	0,4	2,65	1,43	6,15	6,15	-
Caulerpa cylindracea	0,66	1,05	2,62	1,25	6,08	12,23	+
Dictyopteris sp.	0,44	1	2,26	1,29	5,25	17,48	+
Halopteris filicina	0	0,82	2,21	1,12	5,13	22,61	+
Crambe crambe	1	0,58	2,13	1,25	4,93	27,54	-
Peyssonnelia spp.	1,13	1,31	1,84	1,05	4,27	31,81	+
Detritus	0,71	0,78	1,82	1,16	4,22	36,03	+
Axinella damicornis	0,58	0,7	1,63	1,11	3,79	39,82	+
Pseudodistoma cyrnusense	0,51	0,36	1,51	1	3,49	43,31	-
Schizomavella mamillata	0,96	0,93	1,49	1,06	3,45	46,77	-
Palmophyllum crassum	0,43	0,44	1,47	0,99	3,41	50,18	+
Parasmittina sp.	0,5	0,09	1,35	0,75	3,14	53,32	-
Raspaciona aculeata	0,33	0,34	1,28	0,87	2,96	56,29	+
Phorbas tenacior	0,39	0,19	1,27	0,73	2,94	59,23	-
Flabellia petiolata	0,34	0,26	1,27	0,76	2,94	62,16	-
Phorbas topsenti	1,36	1,39	1,26	1,01	2,92	65,08	+
Zanardinia typus	0	0,4	1,07	0,67	2,48	67,56	+
Dictyonella sp.	0,41	0	1,02	0,72	2,37	69,93	-
Carpomitra costata	0	0,35	0,99	0,52	2,3	72,24	+
Turf algae	1,77	1,82	0,92	1,05	2,13	74,37	+
Mesophyllum sp.	1,62	1,82	0,91	1,21	2,11	76,47	+
Orange Sponge 3	0,18	0,23	0,89	0,64	2,07	78,55	+
Cliona viridis	0,24	0,1	0,8	0,56	1,87	80,41	-
Pleraplysilla spinifera/Dysidea avara	0,21	0,08	0,61	0,57	1,42	81,83	-
Rhynchozoon spp.	0,18	0,1	0,6	0,53	1,4	83,23	-
Turf invertebrate	2,01	1,93	0,58	1,27	1,34	84,58	-
Reteporella sp.	0,24	0	0,57	0,49	1,32	85,9	-
Caryophyllia inornata	0,13	0,11	0,55	0,47	1,28	87,18	-
Didemnum sp.1	0,05	0,15	0,47	0,47	1,1	88,28	+
Hoplangia durotrix	0,18	0	0,47	0,42	1,08	89,36	-
Crella pulvinar	0,05	0,15	0,46	0,44	1,07	90,43	+

Supplementary Table S8. Details of GLMM comparing the abundance of *Caulerpa cylindracea* along time and among treatments. Degrees of freedom, F- and p-values for each factor are reported; p-values <0.001 are represented by ***, <0.01 by ** and <0.05 by*.

Factor	Df	F-value	p-value
Year	2	193,09	***
Treatment	2	4,17	*
Year x Treatment	4	3,91	***

Supplementary Table S9. Details of the ANOVA lf the GLMM (Chi square value) for the different diversity values (richness, Shannon's Index and evenness**.** Degrees of freedom, F- and p-values for each factor are reported; p-values <0.001 are represented by ***, <0.01 by ** and <0.05 by*.

Factor	Factor	Df	F-value	p-value
Richness	Year	2	40,65	***
	Treatment	2	2,57	0,08
	Year x Treatment	4	0,35	0,84
Shannon's index	Year	2	1,03	0,36
	Treatment	2	0,02	0,98
	Year x Treatment	4	0,008	1,00
Evenness	Year	2	0,0008	1,00
	Treatment	2	0,02	0,99
	Year x Treatment	4	0,003	1,00
